# Clinical and economic burden of idiopathic pulmonary fibrosis: a retrospective cohort study

**DOI:** 10.1186/s12890-015-0165-1

**Published:** 2016-01-05

**Authors:** Karina Raimundo, Eunice Chang, Michael S. Broder, Kimberly Alexander, James Zazzali, Jeffrey J. Swigris

**Affiliations:** Genentech Inc., DNA Way, South San Francisco, CA 94080 USA; PHAR, LLC, Beverly Hills, CA USA; National Jewish Health, Denver, CO USA

**Keywords:** Idiopathic pulmonary fibrosis, Health care resource utilization, Health care costs

## Abstract

**Background:**

Idiopathic pulmonary fibrosis (IPF) is a devastating condition with a variable course. Not uncommonly, IPF patients are hospitalized for respiratory-related causes, including disease worsening. This study aimed to characterize the prevalence, and economic and health care burden of IPF.

**Methods:**

Retrospective insurance claims data collected yearly between January 1, 2009 and December 31, 2011, were used to determine prevalence and calculate all-cause and respiratory-related resource utilization and costs. Patients had at least one inpatient claim or two outpatient claims for IPF (ICD-9-CM code 516.3). Results for health care burden are reported for the 2011 cohort (similar findings in 2009–2010). Costs are reported in 2011 US dollars ($).

**Results:**

Patients with IPF had a mean age of 69.8–71.3 years. Overall prevalence for IPF was 28.8, 28.1 and 19.8 per 100,000 insured persons in 2009, 2010 and 2011. In each year, prevalence increased with age. In 2011, 37.7 % of patients were hospitalized at least once for any cause; 19.5 % for respiratory-related reasons. Also in 2011, the mean number of all-cause outpatient visits and respiratory-related office visits was 18.5 and 5.7 per patient, respectively. All-cause health care costs in 2011 were $59,379 per patient; 36.6 % of costs ($21,732) were respiratory related.

**Conclusions:**

The prevalence of IPF in this claims database increased with age, with a notable increase in those over 65 years. IPF is associated with a large economic and health care burden. Additional research is needed to determine how such burden might be reduced.

## Background

Idiopathic pulmonary fibrosis (IPF) is a chronic disease in which normal lung parenchyma is replaced with fibrotic tissue, leading to dyspnea, cough and impaired lung function [1]. IPF predominantly occurs in older adults [1] and the progressive nature of the disease results in a median survival time of approximately 3 years from diagnosis [2, 3]. Commonly reported comorbid conditions among IPF patients include pulmonary hypertension, gastroesophageal reflux disease, obesity, emphysema, cardiovascular conditions and obstructive sleep apnea [4, 5]. IPF itself and these common comorbidities significantly impair health-related quality of life among patients [6] and impose a substantial burden on health care resources [5].

Although data are limited, compared with patients without IPF, those with IPF require greater health care resource utilization, have a greater number of comorbidities [7, 8] and generate higher direct medical costs [7]. Consensus guidelines recommend lung transplantation and long-term oxygen as potential therapies for IPF [4]—both are costly treatments. The FDA recently approved pirfenidone and nintedanib for the treatment of IPF; how these therapies affect resource utilization and cost in a real-world setting is yet to be determined [9, 10].

IPF incidence rate and prevalence estimates vary across studies because of differences in design and data sources [11, 12]. The prevalence of IPF ranges from 0.7 – 6.4 per 100,000 individuals in Taiwan to 27.9–63.0 per 100,000 in the USA, while incidence has been reported as 0.6–1.4 and 8.8–17.4 per 100,000 person-years, in Taiwan and the USA, respectively [13]. Because the incidence and prevalence of IPF is greater among older citizens, with the US population aging [12–14], it is expected that the economic burden and health care resource utilization of IPF will likely increase in the foreseeable future. Thus, there is a desperate need for improved understanding of the burden of IPF on health care systems. In this study, we used a commercial insurance claims database to characterize the economic burden of IPF by investigating health care resource utilization and costs associated with patient management.

## Methods

### Data source

Data for this study were obtained from a large Health Insurance Portability and Accountability Act (HIPAA) of 1996-compliant insurance claims database. The database used was the Humedica (from Optum) database and access was via a license agreement (not openly available). The database provides information on medical and pharmacy claims for approximately 30 million lives in the USA. In this study, extracted data covered four geographical regions in the USA (Midwest, Northeast, South and West). In compliance with HIPAA 1996, all data were void of identifying information and therefore the study is exempt from institutional review board approval.

### Inclusion criteria

**Fig. 1:**
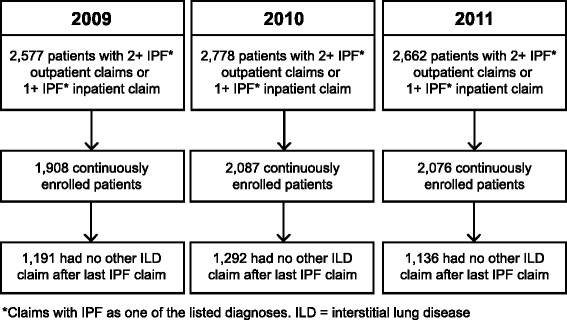
Patient identification

This study included three cohorts, each formed by capturing data spanning a 1 year period (January 1, 2009–December 31, 2009; January 1, 2010–December 31, 2010 and January 1, 2011–December 31, 2011 [Fig. 1]). To be included in a cohort, patients needed at least one inpatient claim or two outpatient claims with IPF as one of the listed diagnosis codes (International Classification of Diseases, Ninth Revision, Clinical Modification [ICD-9-CM] code 516.3) anytime in that calendar year. Patients were required to be continuously enrolled with the health plan in the same calendar year and have no other type of interstitial lung disease (ILD) diagnosis after their last IPF claim in that calendar year.

Some patients may have had an IPF diagnosis for the entire period (and are thus included in more than one of the three cohorts), and other patients may have been diagnosed in the last month of a study period (e.g. December) and were therefore included in the cohort for that entire year. In 2012, the ICD-9 code for IPF changed from 516.3 to 516.31. Due to the possibility of coding errors in 2012 resulting from this change, the study was limited to 2009–2011. For each year, insurance claims were extracted from the database for all patients. Respiratory-related medical claims were classified as those associated with ICD-9-CM codes 460.xx–519.xx.

### Resource utilization and costs

Total health care costs, non-pharmacy costs (including inpatient and outpatient services costs) and pharmacy/medication costs were analyzed for all-cause and respiratory-related health care utilization. The number of inpatient hospitalizations, emergency department (ED) visits and physician office visits was also assessed. Respiratory-related medication costs include all outpatient pharmacy claims of respiratory therapy, oral antibiotics and all possible IPF medication. In these analyses, medication costs were assessed and include any of the eight groups of prescription medications used to possibly treat IPF (corticosteroids, azathioprine, cyclophosphamide, N-acetylcysteine, sildenafil, interferon-gamma, bosentan, etanercept). No FDA-approved treatments were available for IPF at the time of this study. The usual physician specialty for IPF care was defined as the physician specialty with the largest plurality of IPF office visits (office visits with primary diagnosis of IPF) with evaluation and management services. For some patients, there was no office visit meeting that definition (e.g. with a primary IPF diagnosis) and they could not be classified. In other patients, there were visits but the physician specialty was not listed, and they could not be classified either.

### Prevalence estimates

Prevalence estimates for each year were calculated based on the number of patients with an ICD-9-CM code for IPF (516.3) and no other type of ILD after their last IPF claim as the numerator. The number of members continuously enrolled in a given calendar year, regardless of diagnosis, was used as the denominator.

### Statistical analysis

Descriptive statistics were generated for baseline data. Means and standard deviations are reported for continuous variables, and counts and percentages for categorical variables. Economic and health care burden are reported for the 2011 cohort (findings were similar in 2009–2010). Estimated costs are reported in 2011 US dollars ($). The Charlson Comorbidity Index, which includes a total of 22 conditions and can predict 10-year mortality risks [15], was used to evaluate the prognosis for the patient populations, in which a range of comorbid conditions were present. Each condition was assigned a score of one, two, three or six, depending on its association with the risk of dying. Scores were summed to provide a total score predictive of mortality. All analyses were performed using SAS® version 9.4.

Demographics are reported for all 3 years and for prevalence estimates, but only 2011 data were used for the utilization analyses. All figures and tables show data for all 3 years.

## Results

### Demographics

The study population consisted of 1191, 1292 and 1136 IPF patients in 2009, 2010 and 2011, respectively (Fig. 1). Patients’ mean age ranged from 69.8 to 71.3 years, and the population was equally balanced by gender. The majority of patients (39.7–42.3 %) originated from the Southern region of the USA, compared with 12–15 % from the Northeast, 18–23 % from the West, and 24–25 % from the Midwest (Table 1). In each annual cohort, the usual physician specialty providing IPF care was a pulmonologist in approximately half of the population (49.2–50.5 %); the physician specialty providing care was unknown in approximately 30 % of cases. The overall Charlson Comorbidity Index remained constant at 3.2 over the three periods (*P* = 0.917). The study population had a high (mean of six) number of chronic conditions and comorbidities contributing to this score (Table 2), including chronic obstructive pulmonary disease (53.4–56.9 %), cardiovascular conditions (48.1–51.1 %) and bacterial pneumonia (24.9–30.9 %).

**Table 1:** Patient demographics

	2009	2010	2011
	(*N* = 1191)	(*N* = 1292)	(*N* = 1136)
Age, years, mean (SD)	69.8 (11.1)	70.0 (11.4)	71.3 (10.6)
Female, n (%)	600 (50.4)	671 (51.9)	558 (49.1)
Region, n (%)			
Midwest	297 (24.9)	309 (23.9)	279 (24.6)
Northeast	152 (12.8)	160 (12.4)	171 (15.1)
South	473 (39.7)	545 (42.2)	481 (42.3)
West	269 (22.6)	278 (21.5)	205 (18.0)
IPF care specialty^a^, n (%)			
Pulmonologist	586 (49.2)	652 (50.5)	553 (48.7)
Primary care	213 (17.9)	217 (16.8)	187 (16.5)
Other (<2 % each)	61 (5.1)	52 (4.0)	43 (3.8)
Unknown	331 (27.8)	371 (28.7)	353 (31.1)

*IPF* = idiopathic pulmonary fibrosis

^a^IPF care specialty defined as the physician specialty with the largest plurality of IPF office visits with evaluation and management services. For some patients, there was no office visit meeting that definition and others whose physician specialty was not listed, these were classified as unknown

**Table 2:** Diagnosis of comorbidities in patients with IPF

	2009	2010	2011
	(*N* = 1191)	(*N* = 1292)	(*N* = 1136)
CCI, mean (SD)	3.2 (2.7)	3.2 (2.7)	3.2 (2.7)
Number of chronic conditions, mean (SD)	5.9 (2.0)	5.9 (2.0)	5.9 (2.0)
Conditions, n (%)			
COPD (including emphysema)	636 (53.4)	692 (53.6)	646 (56.9)
Cardiovascular conditions	573 (48.1)	626 (48.5)	581 (51.1)
Bacterial pneumonia	364 (30.6)	380 (29.4)	351 (30.9)
Lung cancer	49 (4.1)	39 (3.0)	43 (3.8)
Pulmonary hypertension	101 (8.5)	131 (10.1)	79 (7.0)
GERD	332 (27.9)	378 (29.3)	336 (29.6)
Obstructive sleep apnea	176 (14.8)	199 (15.4)	169 (14.9)
Obesity	95 (8.0)	106 (8.2)	87 (7.7)

*CCI* = Charlson Comorbidity Index, *COPD* = chronic obstructive pulmonary disease, *GERD* = gastroesophageal reflux

Cardiovascular conditions included ischemic heart disease, myocardial infarction, congestive heart failure, pulmonary hypertension

### Prevalence

**Fig. 2:**
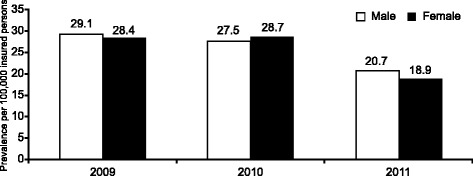
Prevalence of IPF by gender

**Fig. 3:**
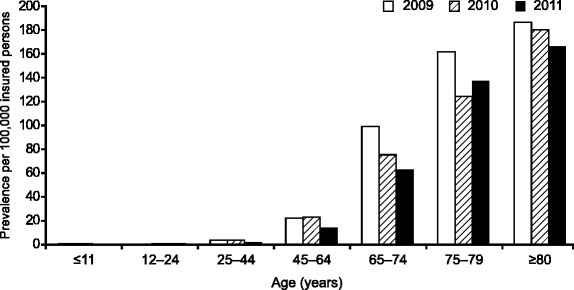
Prevalence of IPF according to age

Overall, IPF prevalence was 28.8 (1191/4,138,796), 28.1 (1292/4,595,629) and 19.8 (1136/5,748,328) per 100,000 insured persons in 2009, 2010 and 2011, respectively. Similar values were observed between genders (Fig. 2). Prevalence increased for patients aged ≥65 years, and the highest prevalence was observed among those ≥80 years old: 185.5 (338/182,231), 179.4 (369/205,708) and 165.9 (347/209,135) per 100,000 insured persons in 2009, 2010 and 2011, respectively; Fig. 3).

### Diagnosis and treatment

In 2011, about half of patients had a diagnostic test performed (either lung biopsy [7.3 %] or computed tomography of the thorax [46.5 %]) consistent with an attempt to confirm a diagnosis of IPF. Use of oxygen at home was observed in 52.6 % of patients; 44.6 % were treated with oral corticosteroids and 1.7 % had a lung transplant. A total of 12.1 % of patients participated in pulmonary rehabilitation.

### Health care resource use and costs

**Fig. 4:**
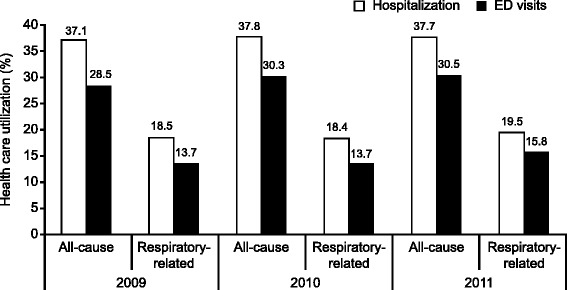
Health care resource utilization according to all-cause or respiratory-related conditions

**Fig. 5:**
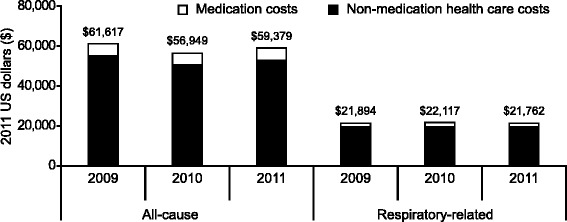
Health care costs (medication and non-medication) due to all-cause or respiratory-related conditions

There was no significant difference over the three periods (*P =* 0.690) in the mean number of all-cause outpatient clinic visits, which in 2011 was 18.5 per patient. Similarly, there was no significant difference over time in the mean number of clinic visits specifically due to respiratory-related conditions (5.7 per patient in 2011, *P* = 0.654). A total of 37.7 % of patients were hospitalized at least once due to any cause, and 7.7 % were hospitalized (for any cause) at least three times. Overall, 30.5 % of patients visited the ED on at least one occasion for any cause. For respiratory-related conditions, 19.5 % of patients (*n* = 221) were hospitalized and 15.8 % (*n* = 179) visited the ED at least once (Fig. 4). The mean annual all-cause health care cost was $59,379 per patient in 2011, with 36.6 % of the total all-cause health care costs spent on respiratory-related care (Fig. 5). Non-medication health care costs (for any condition) were $52,716 per patient. The majority of this was for inpatient ($38,032) rather than outpatient services ($14,684). Non-medication costs for respiratory-related conditions accounted for 37.2 % of health care cost. The main medications costs were for generic corticosteroids, which were used in approximately 60 % of patients, with no significant differences in use over the three periods (*P* = 0.787).

## Discussion

IPF is a severe, chronic condition that primarily affects patients older than 60 years of age, who often have other comorbidities and who require substantial health care resources. In this study, we used a large, de-identified insurance claims database to identify three annual cohorts of IPF patients. The heightened presence of chronic conditions and comorbidities in the population was reflected in the 10-year Charlson Comorbidity Index score of 3.2. Overall, IPF patients were found to be frequent health care service users, with nearly 19 outpatient visits per year. More than one-third of patients were hospitalized—and approximately 30 % visited the ED—at least once per year. During that time period, the provision of inpatient and outpatient care amounted to a total annual cost of approximately $60,000 per patient, with about one-third of this spent on respiratory-related care.

A high level of variability exists in epidemiologic reports of incidence and prevalence in IPF, most likely due to differences in diagnostic testing and case definition, as well as differences in study populations and study design [4, 5, 11]. However, it is generally agreed that prevalence is increasing due to the aging of the population and improved awareness and earlier diagnosis of the condition [13, 14]. In a recent review of 15 studies investigating IPF prevalence in the USA, the authors reported values from 14 to 27.9 cases per 100,000 population [16]. Other data confirm a higher prevalence among patients aged 75 years or older (67.4 per 100,000 persons) compared with people in the 18–34-year age range (0.8 per 100,000 persons) [12]. In our study, overall prevalence ranged from 19.8 to 28.8 per 100,000 insured persons and was lowest in 2011; when data were analyzed over the 5-year age group, prevalence was highest in patients aged 80 years or over (165.9–185.5 per 100,000 insured persons).

However, health care utilization and costs were similar across all 3 years. An increase in the total number of insured people (from 4.1 million in 2009 to 5.7 million in 2011)—the denominator—is likely to have contributed to the lower prevalence of IPF in 2011, since the numbers of IPF patients (the numerator) were similar over the 3-year period. In fact, the higher number of insured people in 2011 was composed of a large number of individuals from the youngest age group (unlikely to have IPF). The number of those aged between 12 and 44 years differed from 1.7 and 1.9 million in 2009 and 2010, to 2.5 million in 2011. Data from the Centers of Disease Control and Progression support our observation, and show that the proportion of uninsured adults aged 19–25 years fell from 35.6 % in the third quarter of 2010 to 27.0 % in the fourth quarter of 2012 [17]. In addition, an analysis of IPF in US Medicare beneficiaries aged 65 years or above between the years 2000–2011 revealed that the incidence of IPF remained stable (overall estimate 93.7 cases per 100,000 person-years), while the prevalence increased from 202.2 to 494.5 cases per 100,000 person-years over this period [18].

The prevalence of IPF is usually reported as higher among males [19–21]; however, we observed no difference by gender. This is possibly due to more women accessing care, but we are unable to verify this supposition. In general, women are more likely to seek health care services than men [22–24], and women are more willing to look after themselves [23]. As in previously published studies, in which IPF prevalence has been reported to increase with age (and the highest rates observed in patients over 75 years old) [16], we observed an increase in IPF prevalence with age in this claims database, with a notable rise in those older than 65 years. We observed that most cases (39.7–42.3 %) derived from the southern area of the USA, a finding that reflects other real-world health care experiences. For example, data from Optum Payer lists, covering over 150 million individuals across all payer types, reveal that 33–46 % of claims are from the Southern area of the US, approximately double those coming from the West, Midwest, and Northeast states [25]. Given the consistency of these observations, we suggest that this phenomenon is likely due to distribution of the source population rather than regional differences in disease prevalence.

Few previous studies have reported on the economic burden of IPF. Most recently, Wu et al. [8] found that IPF patients were more likely to use health care resources than non-IPF patients (number of hospitalizations 0.63 vs. 0.31, emergency room visits 0.62 vs. 0.48 and outpatient visits 5.7 vs. 3.1 per person-year). Similarly, Collard et al. [7] reported that the all-cause hospital admission rate (0.5 per person-year) and outpatient visit rate (28.0 per person-year) for IPF patients were both nearly two-fold higher than in controls. Total direct costs for patients with IPF were approximately $26,000 per person-year. Differences in case-finding and expense-inclusion likely account for differences in cost between that and our study [16]. Rather than limiting expenses to those incurred for IPF alone, to help reflect the true cost of care for these patients, we included costs and health care utilization for any cause and, for other analyses, for any respiratory-related condition (including IPF, bronchitis, pneumonia, etc.). These data reflect actual patient care rather than a clinical trial situation. In clinical practice, patients are often given other diagnoses (e.g. myocardial ischemia, asthma, chronic obstructive pulmonary disease [COPD], obesity, ILD) and are evaluated by more than two practitioners before ultimately being correctly diagnosed with IPF. Some patients in our study may have been initially diagnosed with a condition other than IPF and had the diagnosis changed to IPF after additional evaluation.

Database analyses come with inherent limitations, and use of secondary data, without direct involvement of the patient or physician, may decrease confidence in the conclusions drawn [26, 27]. Insurance claims data depend on professional ICD coding, and, given the complexity of the disease process, it must be recognized that accurate diagnosis of IPF can be challenging [27, 28]. In the clinical setting, some cases may be misdiagnosed, and coding patterns may vary between professionals. In this study, the physician specialty for IPF care was pulmonologist in approximately 50 % of patients, and the other half were seen by a primary health care physician or a physician whose specialty was not recorded, for most of their IPF care visits. It is indeed likely that some patients whose practitioners were recorded as unknown may actually have seen a pulmonologist. Guidelines recommend high-resolution computed tomography (HRCT) of the lungs and/or surgical lung biopsy to diagnose IPF [4]. In this study, the lower than expected number of patients with a diagnostic test is a reflection of the cross-sectional study design; it is likely that patients had a HRCT scan conducted at the time of their diagnosis (e.g. diagnosed with IPF prior to 2009), but this may have been outside the study period.

Furthermore, approximately 50 % of IPF patients also had a diagnosis of COPD, including emphysema; whether these were incorrect diagnoses in patients who previously smoked (but did not have COPD) or correctly coded comorbid conditions cannot be determined from the available data. To help reduce the effect of coding inconsistencies, data on all-cause and respiratory-related conditions were considered. This included individuals with at least 1 inpatient claim or two outpatient claims with IPF as one of the diagnosis codes, in hopes of capturing all patients with IPF, including those who may have been misdiagnosed. To limit miscoding, patients who had a non-IPF ILD claim after the last IPF claim in that calendar year were excluded from the analysis. Similar criteria have been used in previous studies [7, 8, 18].

Because the ICD-9 code for IPF changed from 516.3 to 516.31 in 2012, the current analysis was limited to 2009–2011 to further limit possible coding errors. Other limitations include the cross-sectional study design and the fact that patients were not required to have IPF for the entire calendar year; thus, given that some patients may only have started to incur costs later in the year, the total economic burden of disease may be underestimated. A cross-sectional view of patients with IPF offers a true-to-life picture of what might be expected in any given year in which some patients are diagnosed early and others later in the year.

## Conclusion

IPF presents a large burden on health care utilization resources. Additional research is needed to determine whether and how this burden might be reduced.

## Abbreviation

CCI: Charlson comorbidity index
ED: Emergency department
HIPAA: Health insurance portability and accountability act
ICD-9-CM: International classification of diseases, ninth revision, clinical modification
ILD: Interstitial lung disease
IPF: Idiopathic pulmonary fibrosis
